# A self-activated and protective module enhances the preclinical performance of allogeneic anti-CD70 CAR-T cells

**DOI:** 10.3389/fimmu.2024.1531294

**Published:** 2025-01-17

**Authors:** Zhao Zhang, Lianfeng Zhao, Tinghui Huang, Zhengliang Chen, Yaoyao Zhao, Junqing Liang, Xudong Ao, Xiaoqiong Jia, Lei Kang, Linghui Kong, Qi Jing, Jianhua Hu, Lili Gu, Feiyan Pan, Zhigang Hu, Lingfeng He, Muya Zhou, Jiannan Chen, Zhigang Guo

**Affiliations:** ^1^ Jiangsu Key Laboratory for Molecular and Medical Biotechnology, College of Life Sciences, Nanjing Normal University, Nanjing, China; ^2^ Department of Research and Development, Nanjing Calmhome Cell & Gene Engineering Institute Co., Ltd., Nanjing, China; ^3^ Peking University Cancer Hospital (Inner Mongolia Campus), Affiliated Cancer Hospital of Inner Mongolia Medical University, Hohhot, Inner Mongolia Autonomous Region, China; ^4^ Center of Biotherapy, Jiangsu Province Geriatric Hospital, Nanjing, China

**Keywords:** chimeric antigen receptor T (CAR-T), allogeneic CAR-T, cancer immunotherapy, solid tumor, CD70-positive cancer

## Abstract

**Introduction:**

Allogeneic chimeric antigen receptor T (CAR-T) therapy, also known as universal CAR-T (UCAR-T) therapy, offers broad applicability, high production efficiency, and reduced costs, enabling quicker access for patients. However, clinical application remains limited by challenges such as immune rejection, and issues with potency and durability.

**Methods:**

We first screened a safe and effective anti-CD70 scFv to construct anti-CD70 CAR-T cells. Anti-CD70 UCAR-T cells were then generated by knocking out TRAC, B2M, and HLA-DRA. To address the limitations of UCAR-T therapy, we developed an 'all-in-one' self-activated and protective (SAP) module, integrated into the CAR scaffold. The SAP module consists of the CD47 extracellular domain, a mutant interleukin 7 receptor alpha (IL7Rα) transmembrane domain, and the IL7Rα intracellular domain, designed to protect UCAR-T cells from host immune attacks and enhance their survival.

**Results:**

SAP UCAR-T cells demonstrated significantly reduced immune rejection from the innate immune system, as evidenced by enhanced survival and functionality both *in vitro* and *in vivo*. The modified UCAR-T cells exhibited improved persistence, with no observed safety concerns. Furthermore, SAP UCAR-T cells maintained process stability during scale-up production, indicating the potential for large-scale manufacturing.

**Discussion:**

Our findings highlight the SAP module as a promising strategy for the preclinical development of anti-CD70 UCAR-T, paving the way for an 'off-the-shelf' cell therapy product.

## Introduction

Chimeric antigen receptor T (CAR-T) cell therapy has revolutionized cancer treatment, particularly in hematologic malignancies, by harnessing the patient’s own T cells to target and eliminate cancer cells (1–4). Despite its clinical success, autologous CAR-T therapy faces significant challenges, including an expensive, lengthy, and complex production process, as well as unstable cell sources due to the variable health conditions of patients (5). To overcome these limitations, allogeneic CAR-T cells, which are derived from healthy donors and can be produced at scale and used across multiple patients, have emerged as a promising solution (6).

To avoid graft versus host disease (GVHD) and host versus graft (HVG) reactions, immunogenicity factors including T cell receptor (TCR) and human leukocyte antigens (HLAs) need to be suppressed in allogeneic CAR-T cells, also known as universal CAR-T (UCAR-T) cells (7–10). Strategies such as knocking out related genes, along with the use of lymphodepleting drugs, have been employed (11–14). However, the efficacy of UCAR-T therapy was not as good as expected, especially in solid tumors (15). This maybe because lack of HLA molecules can increase the rejection from natural killer (NK) cells (16). Additionally, these modifications can potentially reduce the viability and anti-tumor efficacy of UCAR-T cells, particularly in the hostile microenvironment of solid tumors.<sup> (17–19)

In this study, CD70 was selected as a therapeutic target for CAR-T therapy due to its high expression in various tumor tissues (20–22). To solve the concerns encountered by UCAR-T therapy, we expressed a self-activated and protective (SAP) module in anti-CD70 UCAR-T cells. We observed that in addition to NK cells, host macrophages caused damage to UCAR-T cells as well. To alleviate the host immune responses upon the infusion of UCAR-T cells, this module incorporates the extracellular domain of CD47, which provides a “don’t eat me” signal to macrophages and NK cells and suppresses innate immune signaling (23, 24). Given the importance of interleukin 7 (IL-7) signaling for the survival and expansion of T cells and CAR-T cells, as well as the generation and maintenance of memory T cells, the transmembrane and intracellular domain of IL-7 receptor alpha (IL7Rα) was included in this SAP module to facilitate the persistence of UCAR-T cells *in vivo* (25–27).

Equipping UCAR-T cells with this dual-function SAP module significantly enhanced their resistance to immune system attacks and improved their survival, persistence, and anti-tumor efficacy within the challenging tumor microenvironment (TME). Furthermore, we demonstrated the process stability of SAP UCAR-T cell production and proved their scalability and clinical applicability. Collectively, our data underscores the preclinical efficacy and safety of SAP UCAR-T cells targeting CD70, paving the way for a promising therapeutic strategy against CD70-positive cancers. In addition, by constructing the SAP module, we proposed a potential method to strengthen the anti-tumor activity of UCAR-T cells.

## Results

### Screening of anti-CD70 CAR and construction of anti-CD70 CAR-T cells

CD70 is a type II transmembrane protein that belongs to the tumor necrosis factor (TNF) family. As an immunoregulatory molecule, it is typically expressed on activated T cells and B cells and functions to promote the proliferation, differentiation, and survival of lymphocytes (28). CD70 is also highly expressed in renal cancer, glioblastoma, and ovarian cancer tissues, as demonstrated by analyses from the Cancer Genome Atlas (TCGA) database (**Supplementary Figure S1A**) and immunohistochemical (IHC) staining results (**Supplementary Figure S1B**), along with findings from previous studies (21, 22, 29). This elevated expression positions CD70 as a promising target for anti-cancer immunotherapy. Therefore, we chose CD70 as the target for constructing CAR-T cells designed to treat solid tumors. We identified tumor cell lines with high CD70 expression, such as U251, ACHN and 786-0, as positive models, while CASKI and BXPC3 served as negative controls for further evaluation (**Supplementary Figure S1C**).

**Figure 1:**
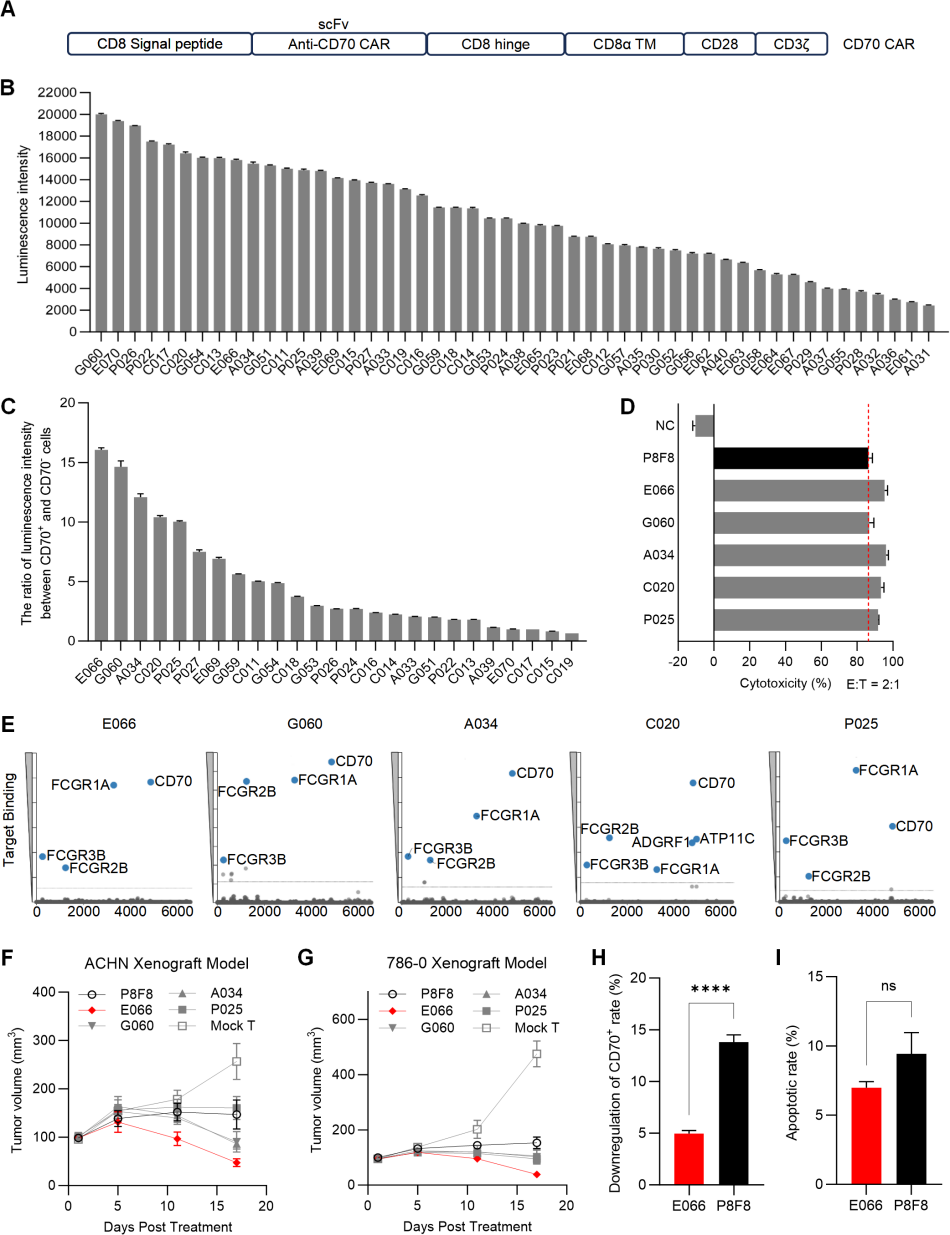
Screening of anti-CD70 CAR sequence **(A)** Schematic diagram illustrating the construction of the anti-CD70 CAR-T. **(B)** Luminescence intensity of CAR-expressing Jurkat reporter cells after co-culturing with 786-0 cells. **(C)** The selected CAR-expressing Jurkat reporter cells from **(B)** were co-cultured with either 786-0 or CD70 knockout 786-0 cells. The luminescence intensity ratio of Jurkat cells co-cultured with the two groups was calculated. **(D)** Cytotoxicity of CAR-T cells obtained from **(C)** was assessed using a short-term killing assay, with P8F8 serving as a positive control. **(E)** Results from membrane protein array (MPA) testing for the CAR sequences selected from **(D)**. **(F, G)** The anti-tumor activity of various CAR-T cells was evaluated in ACHN **(F)** and 786-0 **(G)** tumor xenograft models. **(H, I)** Labeled and unlabeled CAR-T cells were co-cultured, and the downregulation of CD70 positive rate **(H)** and the apoptotic rate **(I)** of the CFSE-labeled CAR-T cells were measured to assess fratricide among the constructed CAR-T cells. Data are presented as means ± SD. Statistical significance was determined using Student’s t-test. ****p < 0.0001; ns, not significant.

To construct anti-CD70 CAR-T cells, single-chain variable fragments (scFvs) targeting the extracellular domain of CD70 were generated by screening a human phage display antibody library in our laboratory, following previously described methods (data not shown) (30). The screened anti-CD70 scFvs were inserted into a CAR structure to create various anti-CD70 CARs (CD70 CARs) (**Figure 1A**).

To evaluate the affinity of CD70 CARs for CD70 on tumor cells, Jurkat reporter cells engineered to express CD70 CARs were co-cultured with CD70-positive 786-0 cells. In the Jurkat reporter cells, a luciferase reporter gene was placed under the control of a nuclear factor of activated T cells (NFAT)-responsive promoter. Upon antigen binding and CAR activation, NFAT would be dephosphorylated, and the NFAT-responsive promoter would be activated to promote the expression of the luciferase reporter gene. The luminescence intensity of the luciferase signal was measured, reflecting the strength of NFAT-mediated signaling and, indirectly, the affinity and functional activity of the CD70 CAR. Jurkat cells expressing the top 25 CD70 CARs based on luminescence intensity were selected for further verification (**Figure 1B**).

To assess the specificity of the CD70 CARs, the CAR-expressing Jurkat reporter cells were co-cultured with both 786-0 cells and CD70 knockout (KO) 786-0 cells. The luminescence intensity was measured, and the ratios of luminescence intensity between the two groups were calculated. The top five CD70 CARs with the highest ratios were then selected for further functional studies (**Figure 1C**).

The top five scFvs selected from **Figure 1C** were then incorporated into CD70 CAR-T cells, and the short-term tumor-killing ability of these CD70 CAR-T cells was assessed by co-culturing them with 786-0 cells for 6 hours. The CAR sequence P8F8, screened by Pfizer Inc. (Patent number: US 2023/0041456 A1), was used as a positive control (29). The results demonstrated that the killing efficiency of all five constructed CAR-T cells were comparable to or even superior to that of P8F8 CAR-T cells (**Figure 1D**).

To evaluate the potential cross-reactivity of the selected scFvs with other membrane proteins, a membrane proteome array (MPA) assay was conducted. The results revealed that, apart from the Fc fragments of IgG receptors (FCGR1A, FCGR2B, and FCGR3B), which served as positive controls, the antibodies constructed with P025, A034, G060, and E066 scFvs exclusively bound to CD70. In contrast, the antibody constructed with the C020 scFv interacted with multiple membrane proteins, including CD70, ADGRF1, and ATP11C (**Figure 1E**). Consequently, the scFvs with better specificity, P025, A034, G060, and E066, were selected for further *in vivo* functionality tests. ACHN and 786-0 xenograft mouse models were established through subcutaneous injection, and the results of CAR-T infusion showed that E066 CAR-T cells outperformed the other CAR-T cells in tumor elimination in both models (**Figures 1F, G**).

Since CD70 is also expressed on activated T cells, CD70 CAR-T cells may recognize and target CD70 on these activated T cells, potentially leading to fratricide (31, 32). To assess fratricide among the generated CD70 CAR-T cells, we conducted a co-culture assay using CFSE-labeled non-CD70-targeting CAR-T cells as target cells and unlabeled CD70 CAR-T cells as effector cells, and the effector-target ratio was set as 1:1. The expression of CD70 on CFSE-labeled cells was measured by flow cytometry before and after co-culture. In this assay, a reduction in CD70-positive cells would indicate fratricide. Cells co-cultured with E066 CAR-T cells exhibited a 5% reduction in CD70 positivity, which was notably lower than the reduction observed in cells co-cultured with P8F8 CAR-T cells (**Figure 1H**). Additionally, the apoptotic rate of CFSE-labeled cells after co-culture with E066 CAR-T cells was approximately 7%, slightly lower than the rate observed in cells co-cultured with P8F8 CAR-T cells (**Figure 1I**). These results indicate that E066 CAR-T cells exhibit minimal fratricide compared to P8F8 CAR-T cells. Consequently, the E066 scFv was selected, leading to the construction of CD70 CAR-T cells with high specificity, strong anti-tumor efficacy, and fratricide-resistance.

### Generation of CD70 UCAR-T cells through triple knockout

**Figure 2:**
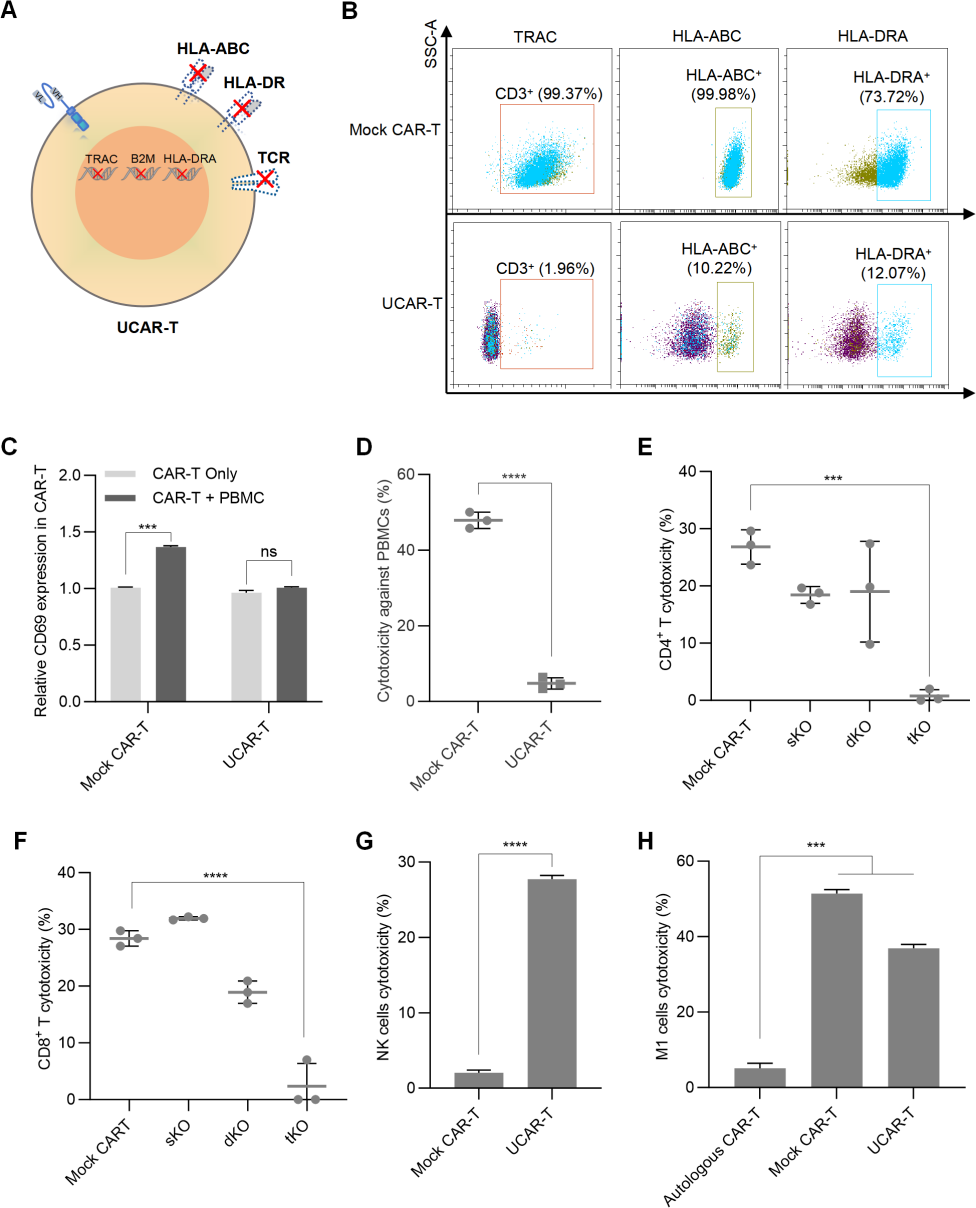
Construction of UCAR-T cells and validation of GVHD and HVG reactions **(A)** Schematic representation of the CAR-T cell knockout method. **(B)** Representative flow cytometric profiles illustrating the knockout efficiency of the triple knockouts in UCAR-T cells. **(C)** Relative CD69 expression in unmodified allogeneic CAR-T (Mock CAR-T) and UCAR-T cells after co-culturing with PBMCs. The levels of CD69 were normalized to that in CAR-T cells without co-culture. **(D)** Cytotoxicity of unmodified allogeneic CAR-T (Mock CAR-T) and UCAR-T cells against PBMCs. **(E–H)** Cytotoxicity of host CD4^+^ **(E)** and CD8^+^ T cells **(F)**, NK cells **(G)**, and macrophages **(H)** against various CAR-T cells. sKO refers to CAR-T cells with a single knockout (TRAC KO); dKO refers to CAR-T cells with double knockouts (TRAC and B2M KO); tKO refers to CAR-T cells with triple knockouts (TRAC, B2M, and HLA-DRA KO), also referred to as UCAR-T. Data are presented as means ± SD from at least three donors. Statistical significance was determined using two-way ANOVA **(C)**, Student’s t-test **(D, G)** and one-way ANOVA **(E, F, H)**. ***p < 0.001, ****p < 0.0001; ns, not significant.

Given the limitations of autologous CAR-T therapy, such as high costs and instability, allogeneic CAR-T cells are being developed. The main challenges associated with allogeneic CAR-T therapy include graft-versus-host disease (GVHD) and host-versus-graft (HVG) reactions, which arise from the TCR and HLA molecules present on the surface of CAR-T cells (7). To inhibit TCR expression, we knocked out the TCR-encoding gene TRAC using CRISPR/Cas9 technology, while the levels of HLA molecules were reduced by silencing B2M and HLA-DRA (**Figure 2A**). For each gene, 10 single-guide RNAs (sgRNAs) were designed, and the knockout efficiency was assessed using flow cytometry analysis (**Supplementary Figure S2A**). The sgRNA with the highest knockout efficiency for each gene was selected.

The T7 endonuclease I (T7E1) assay was also conducted to assess the editing efficiency achieved by the CRISPR/Cas9 system. T7E1 specifically cleaves mismatched DNA strands, enabling the identification of bands corresponding to edited and unedited alleles. The editing efficiency was quantified by comparing the intensity of the cleaved and uncut products. The results indicated successful knockout of TRAC, B2M, and HLA-DRA, with efficiencies of 70.4%, 74.2%, and 66.3%, respectively (**Supplementary Figure S2B**).

To evaluate the off-target risks associated with the sgRNAs used and ensure the safety of the knockout system, oligodeoxynucleotide (ODN) tags were integrated into CAR-T cells, followed by high-throughput sequencing of the PCR-amplified DNA fragments. The off-target rates for these sgRNAs were found to be 8.97%, 2.70%, and 0% for TRAC, B2M, and HLA-DRA, respectively (**Supplementary Figure S2C**). Subsequently, we performed a triple knockout (tKO) of TRAC, B2M, and HLA-DRA in CD70 CAR-T cells to generate CD70 UCAR-T cells, achieving knockout efficiencies of over 80% for each gene (**Figure 2B**).

### GVHD and HVG reactions were less frequently observed in UCAR-T cells

To examine the GVHD induced by UCAR-T cells, we co-cultured UCAR-T cells and unmodified CAR-T cells (referred to as mock CAR-T cells) with sublethally irradiated human peripheral blood mononuclear cells (PBMCs) obtained from a different donor. Compared to mock CAR-T cells, UCAR-T cells exhibited less activation upon PBMC stimulation, as indicated by changes in CD69 expression (**Supplementary Figure S3A** and **Figure 2C**). The cytotoxicity of the allogeneic CAR-T cells against PBMCs was assessed by measuring the apoptotic rate of PBMCs. Mock CAR-T cells demonstrated a significantly higher killing rate against PBMCs, while UCAR-T cells induced minimal killing (**Figure 2D**). These results indicate that the GVHD potential of UCAR-T cells has been reduced due to the knockout of TCR in CAR-T cells.

Mock CAR-T cells, TRAC single knockout CAR-T cells (sKO), TRAC and B2M double knockout CAR-T cells (dKO), and TRAC, B2M, and HLA-DRA triple knockout CAR-T cells (tKO) were co-cultured with host CD4^+^ and CD8^+^ T cells to assess HVG reactions. Activation of host T cells was evaluated by measuring CD69 levels. Both CD4^+^ and CD8^+^ T cells co-cultured with UCAR-T cells exhibited less activation compared to the other groups (**Supplementary Figures S3B, E**). Additionally, host T cells demonstrated lower cytotoxicity against UCAR-T cells, indicating a significant reduction in the risk of triggering HVG responses (**Figures 2E, F**).

In addition to T cells, host NK cells and macrophages can also target allogeneic CAR-T cells. We observed that when UCAR-T cells were co-cultured with NK cells, their apoptosis increased due to the knockout of HLA-I (**Figure 2G**), consistent with previous studies (16). Furthermore, while macrophages induced less damage to UCAR-T cells compared to mock CAR-T cells due to the knockouts of HLA molecules, their cytotoxicity was still significantly higher than that observed in autologous CAR-T cells derived from the same donor as the macrophages (**Figure 2H**).

### CD47 overexpression protected UCAR-T cells from the attacks of NK cells and macrophages

**Figure 3:**
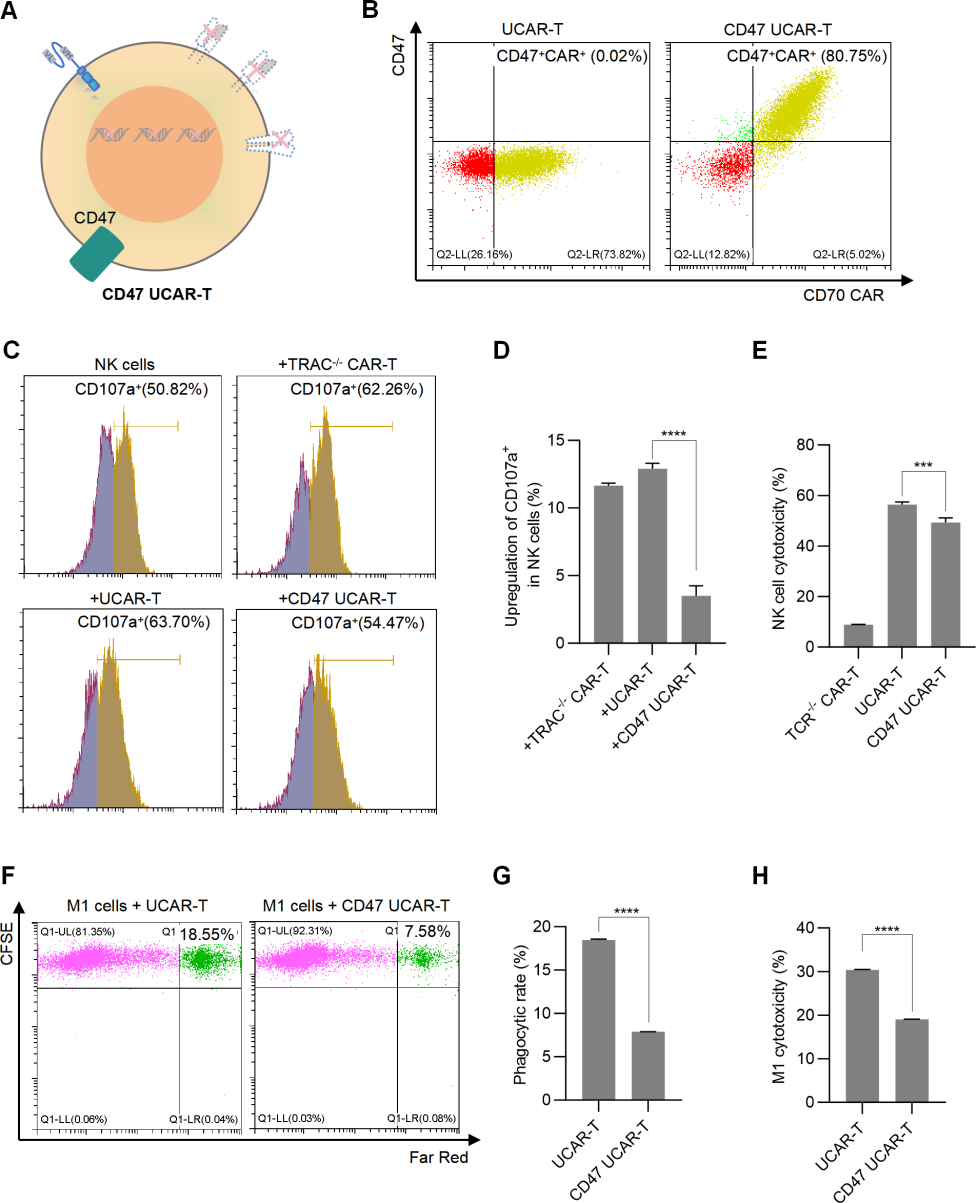
Overexpressing CD47 in UCAR-T Cells reduced NK cell and macrophage-mediated immune rejection **(A)** Illustration of CD47 UCAR-T cells. **(B)** Representative flow cytometric profiles of CD47 and CAR expression on CD47 UCAR-T cells. **(C)** Representative flow cytometric profiles showing CD107a expression on NK cells with or without co-culture with TCR-/- CAR-T, UCAR-T, and CD47 UCAR-T cells. **(D)** Upregulation of CD107a levels of NK cells following co-culture with various CAR-T cells. **(E)** Cytotoxicity of NK cells against various CAR-T cells, determined by comparing cell numbers before and after co-culture. **(F)** Representative flow cytometric profiles illustrating fluorescence expression on cells, where dual fluorescent signals indicate phagocytosis by M1 macrophages. **(G)** Phagocytosis rate of macrophages on UCAR-T cells and CD47 UCAR-T cells. **(H)** Cytotoxicity of macrophages against UCAR-T cells and CD47 UCAR-T cells. Data are means ± SD from at least 3 donors. Statistical significance was determined by one-way ANOVA **(D, E)** and Student’s t-test **(G, H)**. ***p < 0.001, ****p < 0.0001.

Since CD47 has been reported to facilitate the immune escape from macrophages and NK cells and protect CAR-T cells (33–35), we overexpressed CD47 in UCAR-T cells and constructed CD47 UCAR-T cells, aiming to reduce HVG reactions of UCAR-T cells (**Figure 3A**). The expressions of the CD70 CAR and CD47 were validated through flow cytometry analysis (**Figure 3B**). Subsequently, the CAR-T cells were co-cultured with NK cells, and NK cell activation, indicated by the upregulation of CD107a, as well as CAR-T cell apoptosis, were measured. The results showed that compared to TRAC KO CAR-T cells, the knockout of HLA molecules in UCAR-T cells led to increased NK cell activation, while the expression of CD47 mitigated this increase (**Figures 3C, D**). Consistently, upon co-culture with NK cells, the apoptotic rate of CD47 UCAR-T cells was lower than that of UCAR-T cells, suggesting that CD47, acting as an inhibitory ligand, reduced NK cell reactivity (**Figure 3E**).

Similarly, to assess whether CD47 UCAR-T cells can withstand macrophage killing, macrophages and UCAR-T cells were labeled with different fluorescent dyes and co-cultured at an effector-to-target (E: T) ratio of 1:1. Following this, cells were analyzed using flow cytometry. A lower percentage of dual-fluorescent cells in the CD47 UCAR-T group indicated a reduced phagocytic rate of macrophages (**Figures 3F, G**). Additionally, a significantly decreased apoptotic rate of CD47 UCAR-T cells was observed, highlighting the crucial role of CD47 in protecting UCAR-T cells from macrophage attacks (**Figure 3H**).

### The SAP module protected UCAR-T cells from immune rejection and enhanced their persistence

**Figure 4:**
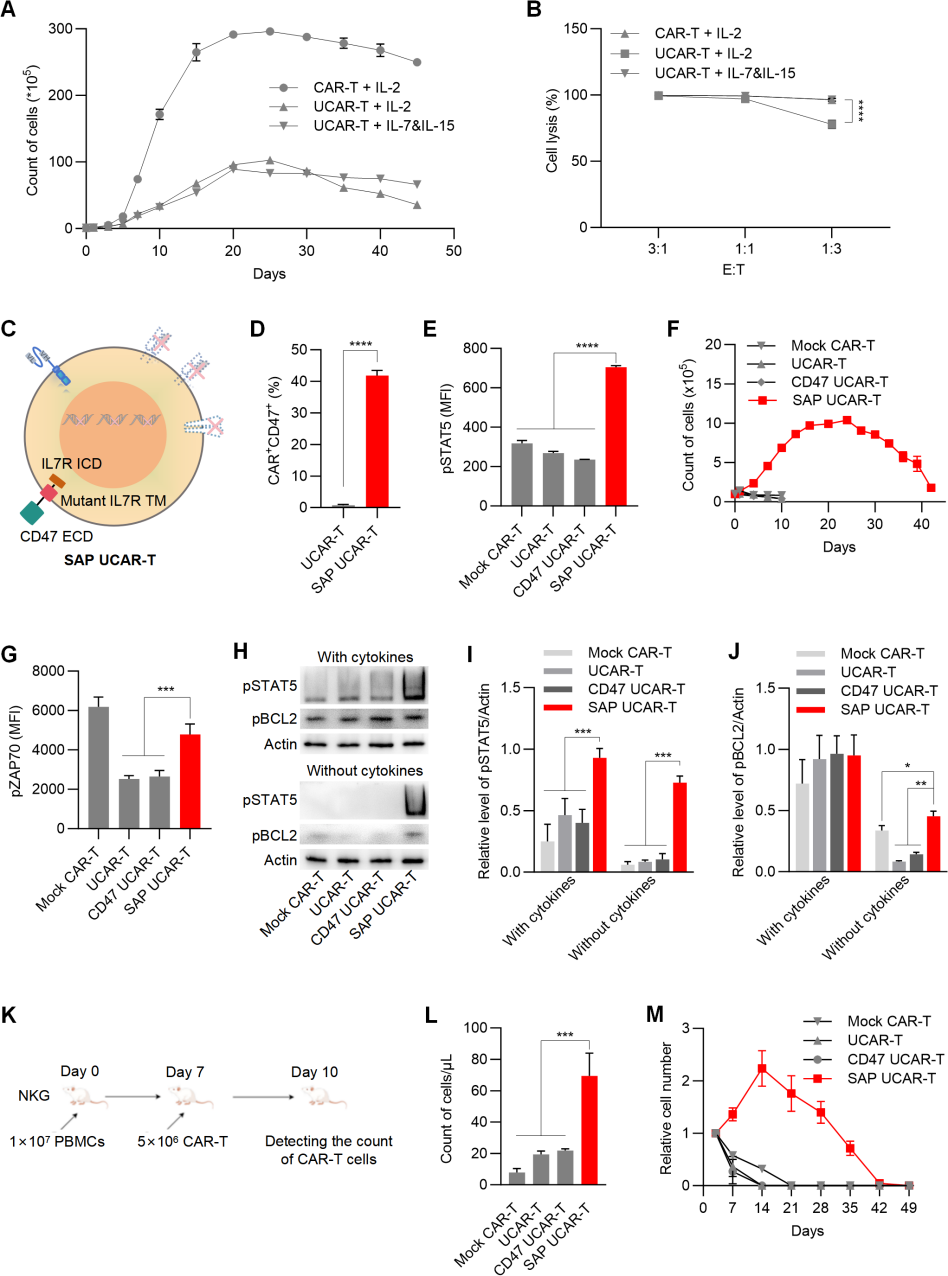
SAP UCAR-T Cells exhibited enhanced resistance to immune rejection and improved persistence both *in vitro* and *in vivo* **(A)** Cell counts of CAR-T and UCAR-T cells cultured with supplements of IL-2 or IL-7&IL-15. **(B)** Cytotoxicity of mock CAR-T cells and UCAR-T cells against 786-0 cells at the indicated E:T ratios. **(C)** Illustration of SAP UCAR-T cells. **(D)** Percentage of CD47^+^CAR^+^ cells in UCAR-T and SAP UCAR-T cells. **(E)** Flow cytometric detection of pSTAT5 levels. **(F)** Proliferation of CAR-T, UCAR-T, CD47 UCAR-T, and SAP UCAR-T cells in the absence of cytokines. **(G)** Flow cytometric detection of pZAP70 levels. **(H)** Levels of pSTAT5 and pBCL2 in various CAR-T cells cultured with or without cytokines, detected by Western blotting. **(I, J)** Quantification of Western blotting results. **(K)** Mice were first injected with human PBMCs, followed by the infusion of CAR-T cells. The quantity of CAR-T cells in the mouse blood was measured three days later. **(L)** Cell counts of CAR-T, UCAR-T, CD47 UCAR-T, and SAP UCAR-T cells in the mice from **(K)**. **(M)** CAR-T cells were injected into non-tumor-bearing mice, and the quantity of CAR-T cells in the mouse blood was continuously monitored. Cell numbers in the blood of mice were normalized to those detected on the first day. Data are presented as means ± SD from at least three donors. Statistical significance was determined by two-way ANOVA **(B, I, J)**, Student’s t-test **(D)** and one-way ANOVA **(G, L)**. *p < 0.05, **p < 0.01, ***p < 0.001, ****p < 0.0001.

Another common concern regarding the application of UCAR-T cells is their inadequate *in vivo* survival and persistence resulting from gene knockouts (17). During *in vitro* expansion, we observed that the survival and proliferation ability of UCAR-T cells were significantly restricted compared to mock CAR-T cells (**Figure 4A**), as was their tumor-killing capability at a lower E:T ratio (**Figure 4B**). Additionally, replacing IL-2 with IL-7 and IL-15 in the culture media showed beneficial effects for the long-term survival and anti-tumor function of UCAR-T cells.

Considering that the extracellular domain of IL-15 closely resembles that of IL-2, we propose that enhancing IL-7 signaling in UCAR-T cells may facilitate the expansion and function of UCAR-T cells. To activate IL-7 signaling independently of IL-7, we introduced a mutant form of the IL-7 receptor (IL-7R) transmembrane domain, which has been reported to drive constitutive signaling via JAK1 (36, 37), along with the intracellular domain of IL-7R into UCAR-T cells. Together with the extracellular domain of CD47, we constructed and expressed a self-activated and protective (SAP) module in UCAR-T cells to generate SAP UCAR-T cells (**Figure 4C**).

The expressions of the CD70 CAR and CD47 were validated using flow cytometry analysis (**Figure 4D**). The activation of IL-7 signaling was confirmed by the upregulation of phosphorylated STAT5 (pSTAT5), a downstream marker of IL-7R signaling (**Figure 4E**). We then assessed the expansion capabilities of various modified CAR-T cells. Without cytokine stimulation, mock CAR-T, UCAR-T, and CD47 UCAR-T cells failed to expand, while SAP UCAR-T cells retained substantial expansion capability (**Figure 4F**).

The upregulation of pro-survival and proliferation molecules was also observed in SAP UCAR-T cells. ZAP70 is a key molecule involved in T cell survival and proliferation (38, 39), and flow cytometry analysis showed that the level of pZAP70 in UCAR-T cells was reduced due to TCR knockout, subsequently inhibiting the function of UCAR-T cells (**Figure 4G**). Following the expression of the SAP module, the level of pZAP70 was upregulated in SAP UCAR-T cells. Additionally, Western blotting results showed significantly elevated levels of activated STAT5 and BCL2 in SAP UCAR-T cells under cytokine-free conditions (**Figures 4H–J**).

Next, NKG mice were injected with human PBMCs to mimic the human immune system, after which CAR-T cells were infused (**Figure 4K**). Compared to mock CAR-T, UCAR-T, and CD47 UCAR-T cells, the number of SAP UCAR-T cells in the blood was significantly higher, indicating reduced HVG reactions and enhanced persistence (**Figure 4L**). Additionally, CAR-T cells were infused into tumor-free immunodeficient mice to examine their *in vivo* survival and proliferation in a stimulation-free environment. It was observed that SAP UCAR-T cells expanded for approximately 14 days and remained detectable until Day 42. In contrast, CAR-T cells lacking the SAP module could not proliferate, and their numbers rapidly declined after infusion (**Figure 4M**). In summary, SAP UCAR-T cells with low immunogenicity, resistance to immune rejection, and superior survival and proliferation capabilities were successfully constructed both *in vitro* and *in vivo*.

### SAP UCAR-T cells demonstrate enhanced anti-tumor efficacy and persistence *in vitro*

**Figure 5:**
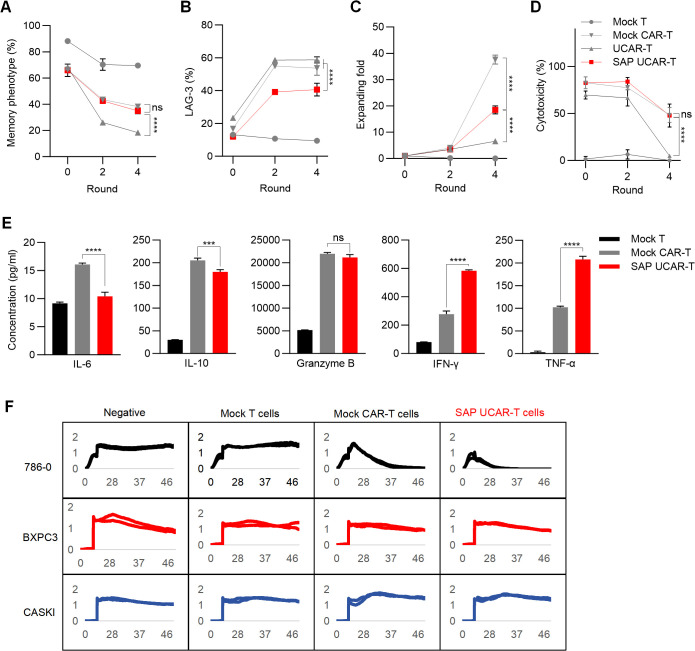
Validation of anti-tumor efficacy function and safety of SAP UCAR-T cells *in vitro* **(A–D)** CAR-T cells were stimulated with tumor cells (R0) and rechallenged for four rounds (R1-R4). The percentages of CAR-T cells exhibiting a memory phenotype **(A)**, along with changes in LAG-3 levels **(B)**, cell proliferation capacity **(C)**, and cytotoxicity **(D)** were assessed after stimulation (R0), following 2 rounds (R2), and after 4 rounds (R4) of tumor cell rechallenge. **(E)** Levels of cytokines released by R2 CAR-T cells were measured. **(F)** Cytotoxicity of SAP UCAR-T cells against 786-0, BXPC3, and CASKI cells was evaluated using a real-time cytotoxicity assay. Data are presented as means ± SD from at least three donors. Statistical significance was determined by two-way ANOVA **(A–D)** and one-way ANOVA **(E)**. ***p < 0.001, ****p < 0.0001; ns, not significant.

To investigate the anti-tumor function of SAP UCAR-T cells *in vitro*, the cells were repeatedly challenged with tumor cells for four rounds. Flow cytometry analysis revealed that the expression of the SAP module significantly increased the memory phenotypes in UCAR-T cells (**Figure 5A**). SAP UCAR-T cells also exhibited the lowest levels of exhaustion among the four groups, as indicated by LAG-3 expression (**Figure 5B**). Furthermore, SAP UCAR-T cells demonstrated enhanced proliferation during this process (**Figure 5C**). In terms of anti-tumor efficacy, during the sustained tumor-killing period, SAP UCAR-T cells consistently displayed strong tumor-killing capabilities, comparable to those of mock CAR-T cells (**Figure 5D**). The SAP module also inhibited the release of the immunosuppressive factor IL-10, and promoted the release of tumor necrosis factor-α (TNF-α) and interferon-γ (IFN-γ) in UCAR-T cells to target tumor cells, while maintaining comparable levels of granzyme B release to those observed in the mock CAR-T group (**Figure 5E**). In addition, elevated IL-6 levels are considered as the hallmark of cytokine-release syndrome (CRS) (40), a common side effect of CAR-T therapy, and the reduced IL-6 releasing observed in SAP UCAR-T cells suggested a lower risk of safety issues (**Figure 5E**).

To evaluate the specificity of SAP UCAR-T cells, mock T cells, mock CAR-T cells, and SAP UCAR-T cells were co-cultured with CD70-positive 786-0 cells, as well as CD70-negative CASKI and BXPC3 cells. Cytotoxicity was assessed using a real-time cytotoxicity assay. The results demonstrated that SAP UCAR-T cells effectively killed only CD70-positive 786-0 cells, exhibiting low off-target cytotoxicity (**Figure 5F**). Collectively, SAP UCAR-T cells showed improved expansion, persistence, and anti-tumor activity *in vitro*, and further *in vivo* validation is required.

### SAP UCAR-T cells show enhanced tumor-killing ability *in vivo*

**Figure 6:**
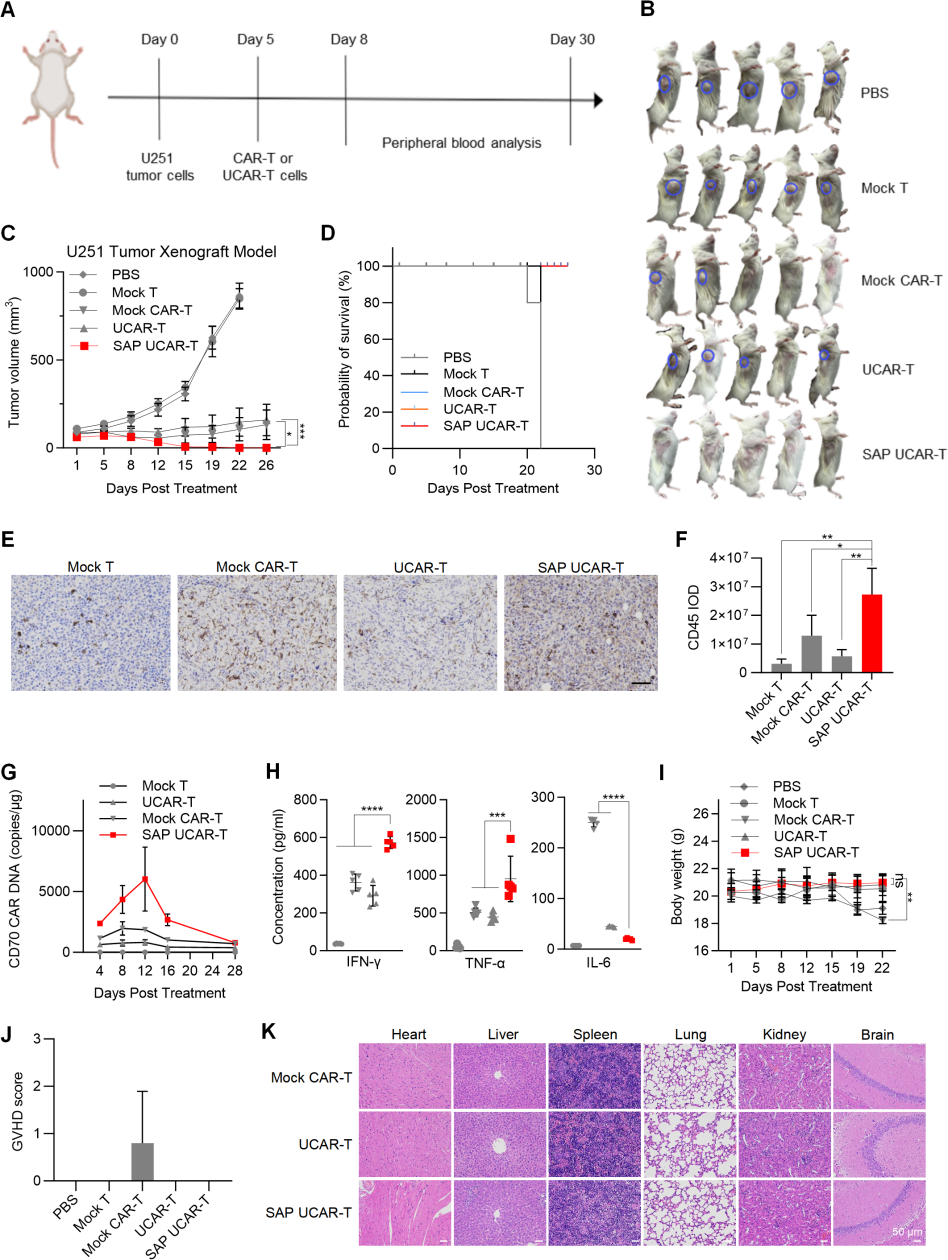
*In vivo* verification of tumor-killing ability and safety of SAP UCAR-T cells **(A)** Mice bearing U251 tumors received injections of PBS, 2×10^6^ mock T cells, mock CAR-T cells, UCAR-T cells, or SAP UCAR-T cells, with continuous monitoring of tumor volumes and body weights. **(B)** Representative images of mice at the end of the experiment. **(C)** Tumor volumes across different groups of mice. **(D)** Survival curves of mice in each treatment group. **(E)** IHC staining of human CD45 in tumors to assess CAR-T cell infiltration. Scale bar, 100 μm. **(F)** Quantification results of CD45 integrated optical density (IOD). **(G)** Quantification of CAR DNA copy numbers in the blood of mice, determined by qPCR. **(H)** Levels of IFN-γ, TNF-α, and IL-6 released into the blood of mice. **(I)** Changes in body weights of mice throughout the study. **(J)** GVHD scores for mice, determined using the clinical GVHD scoring criteria established by the NIH. **(K)** H&E staining to evaluate tissue damage caused by CAR-T cells in mice. Scale bar, 50 μm. Data are presented as means ± SD. Statistical significance was determined by two-way ANOVA **(C, I)** and one-way ANOVA **(F, H)**. *p < 0.05, **p < 0.01, ***p < 0.001, ****p < 0.0001; ns, not significant.

To validate the anti-tumor activity of SAP UCAR-T cells *in vivo*, a U251 tumor xenograft mouse model was employed. When the tumor volume reached approximately 100 mm³, mice were injected with PBS or 2×10^6^ mock T cells, mock CAR-T cells, UCAR-T cells, or SAP UCAR-T cells (**Figure 6A**). While mock CAR-T and UCAR-T cells significantly inhibited tumor growth, only the SAP UCAR-T group achieved 100% tumor clearance (**Figures 6B, C**). Additionally, the treatment with CAR-T cells prolonged the survival of mice (**Figure 6D**). IHC staining for CD45 in tumor tissues revealed that SAP UCAR-T cells exhibited a stronger infiltration ability into the tumor environment (**Figures 6E, F**).

Mice blood was collected on Day 4, 8, 12, 16 and 28, and real-time quantitative PCR results demonstrated a significantly higher number of CD70 CAR copies in the SAP UCAR-T group (**Figure 6G**). Additionally, cytokine levels in the blood were assessed, revealing that both IFN-γ and TNF-α levels were elevated in the SAP UCAR-T group compared to the mock CAR-T and UCAR-T groups. Conversely, the level of the inhibitory cytokine IL-6 was lower in the SAP UCAR-T group (**Figure 6H**).

### *In vivo* safety assessment of SAP UCAR-T cells

A slight weight loss occurred in the mock CAR-T group, potentially due to GVHD and HVG reactions, while no significant changes in mouse weight were observed in other groups (**Figures 6I**). Throughout the CAR-T treatment period, various clinical parameters—such as skin condition, fur quality, body weight, posture, and survival—were statistically analyzed according to the NIH (National Institutes of Health) clinical GVHD scoring criteria (41). The scoring results indicated that neither SAP UCAR-T nor UCAR-T cells produced any noticeable GVHD reactions (**Figure 6J**). Additionally, to assess the toxicity of CAR-T cells on other mouse tissues, hematoxylin and eosin (H&E) staining was performed, revealing no damage to the major tissues in the mice treated with CAR-T cells (**Figure 6K**).

To further assess the safety of SAP UCAR-T cells *in vivo*, up to 6×10^6^ cells were injected into 786-0 tumor-bearing mice. The successful elimination of tumors confirmed the efficacy of SAP UCAR-T cells in this mouse model (**Supplementary Figure S4A**). Monitoring of body weight (**Supplementary Figure S4B**) and H&E staining of major tissues (**Supplementary Figure S4C**) indicated that the high-dose injection of SAP UCAR-T cells did not raise any safety concerns. Additionally, multiple doses (up to five doses) of SAP UCAR-T cells were administered to the same tumor-bearing mice (**Supplementary Figure S4D**). The results demonstrated effective tumor clearance (**Supplementary Figure S4E**), with no body weight loss or tissue damage observed, even after five doses of injection (**Supplementary Figures S4F, G**). Collectively, these findings suggest that SAP UCAR-T cells possess enhanced tumor-killing ability *in vivo* without inducing GVHD or causing tissue damage in mice.

### The SAP module modulated the transcriptional profiles of UCAR-T cells

**Figure 7:**
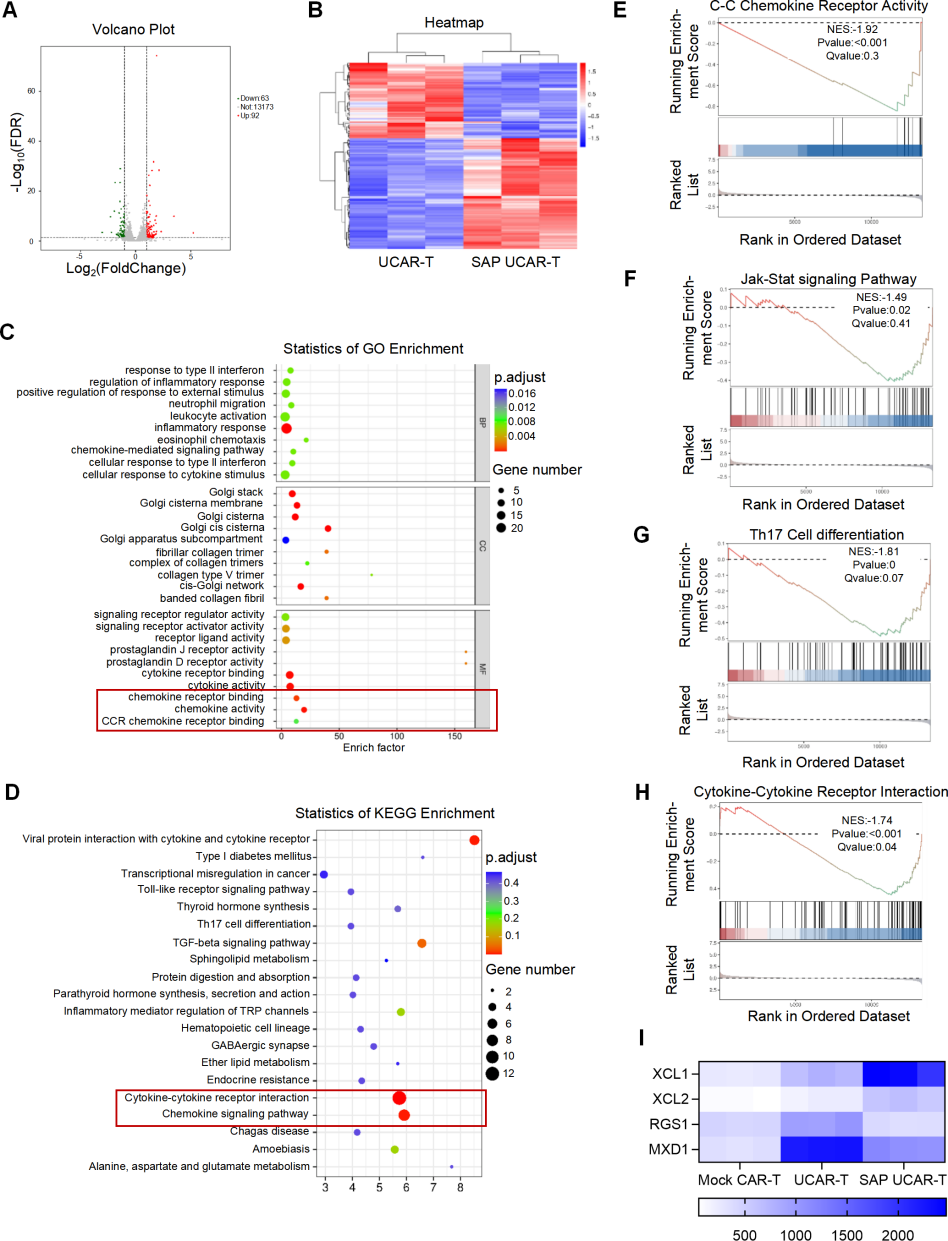
Upregulation of pro-persistence and functionality genes in UCAR-T cells by the SAP module **(A)** Volcano plot illustrating differentially expressed genes (DEGs) in SAP UCAR-T cells compared to UCAR-T cells. **(B)** Heatmap depicting the cluster analysis of DEGs. **(C)** Gene Ontology (GO) analysis of DEGs. **(D)** Kyoto Encyclopedia of Genes and Genomes (KEGG) analysis of DEGs. **(E)** Gene Set Enrichment Analysis (GSEA) GO analysis showing significant upregulation of C-C chemokine receptor activity genes in SAP UCAR-T cells relative to UCAR-T cells. **(F-H)** GSEA KEGG analysis indicating significant upregulation of Jak-Stat signaling pathway **(F)**, Th17 cell differentiation genes **(G)** and Cytokine-cytokine receptor interaction **(H)** in SAP UCAR-T cells compared to UCAR-T cells. **(I)** Heatmap illustrating significant changes in the expression levels of genes XCL1, XCL2, RGS1, and MXD1 among CAR-T, UCAR-T, and SAP UCAR-T cells.

To investigate how the SAP module promoted survival, proliferation, and anti-tumor function of UCAR-T cells, RNA sequencing was conducted on mock CAR-T, UCAR-T, and SAP UCAR-T cells. Both volcano plot and cluster analysis revealed significant differences in gene expression between UCAR-T cells and SAP UCAR-T cells, with 92 genes upregulated and 63 genes downregulated in SAP UCAR-T cells (**Figures 7A, B**). KEGG and GO enrichment analyses indicated alterations in lymphocyte reactivity and chemokine signaling pathways attributable to the SAP module (**Figures 7C, D**). Gene set enrichment analysis (GSEA) demonstrated that C-C chemokine receptor activity, JAK-STAT signaling pathways, Th17 cell differentiation and cytokine-cytokine interaction-related genes were significantly upregulated in SAP UCAR-T cells (**Figures 7E–H**).

Notably, X-C motif chemokine ligand 1 and 2 (XCL1 and XCL2), which play a role in leukocyte activation (42, 43), were significantly elevated in SAP UCAR-T cells. In contrast, regulator of G protein signaling 1 (RGS1) and MAX dimerization protein 1 (MXD1), considered as negative regulators of T cell survival, expansion and function (44, 45), were downregulated in these cells (**Figure 7I**).

### Scale-up production demonstrated the process stability of SAP UCAR-T cells

**Figure 8:**
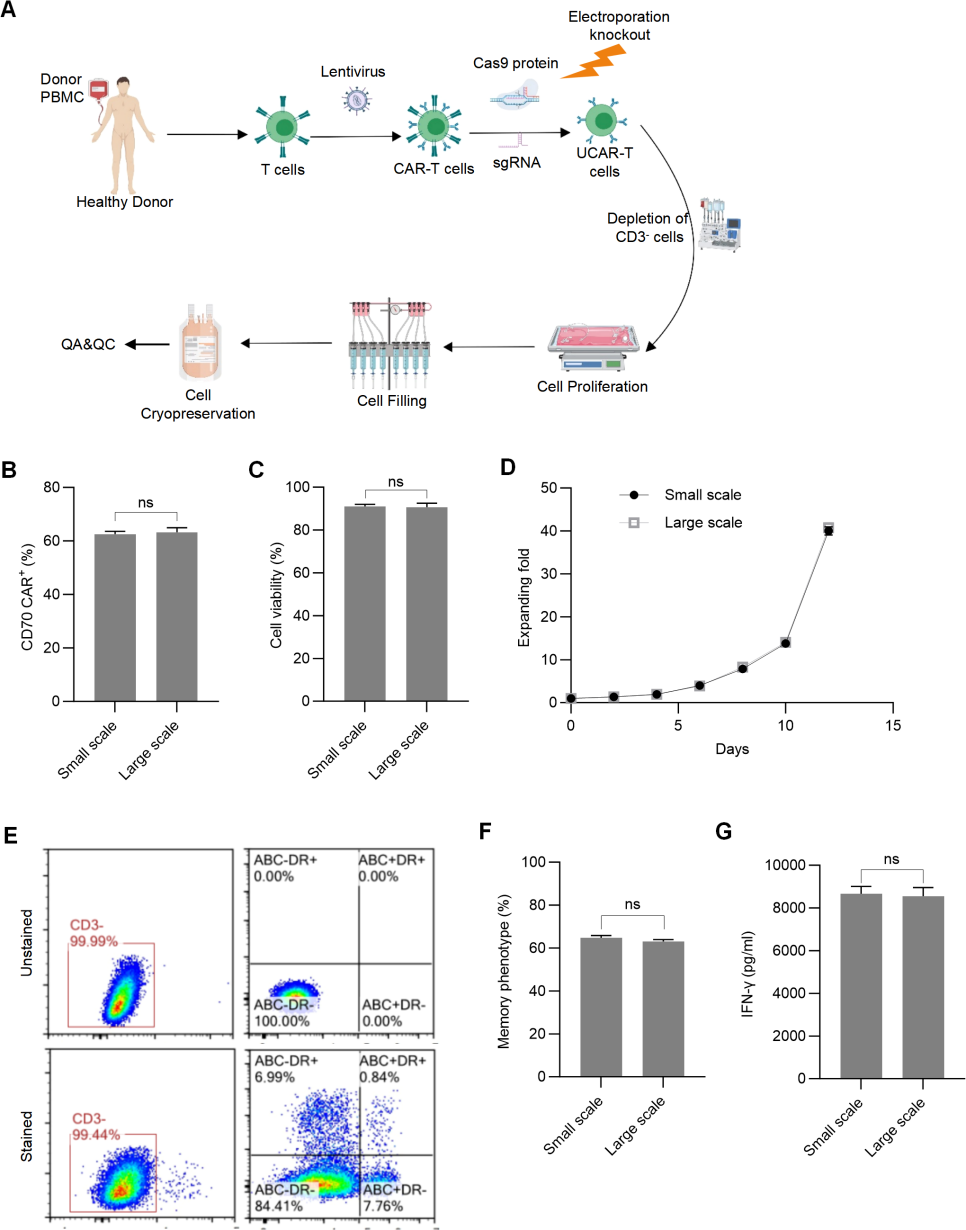
Validation of manufacturing stability for SAP UCAR-T cells through large-scale production **(A)** Overview of the manufacturing process for SAP UCAR-T cells intended for clinical use. **(B)** Assessment of the CAR positive rate in produced cells. **(C)** Measurement of cell viability. **(D)** Evaluation of expanding folds of SAP UCAR-T cells generated from small scale and large scale production. **(E)** Representative flow cytometric profiles demonstrating knockout efficiency in SAP UCAR-T cells. **(F)** Comparison of the percentage of memory phenotype in SAP UCAR-T cells produced in small-scale and large-scale production. **(G)** Measurement of IFN-γ levels secreted by SAP UCAR-T cells from both small-and large-scale production. Data are presented as means ± SD from 3 donors. Statistical significance was determined by Student’s t-test. ns, not significant.

A complete scale-up workflow was established to evaluate the potential of engineered SAP UCAR-T cells for clinical application (**Figure 8A**), and scale-up production of SAP UCAR-T cells was accomplished by OBiO Technology (Shanghai). In detail, T cells were isolated from the PBMCs of healthy donors and activated using CD3/CD28 magnetic beads. One day post-activation, the activated T cells were transduced with lentivirus, followed by electroporation for gene knockout using the CRISPR/Cas9 system. Residual CD3^+^ T cells were then removed using CD3 positive selection magnetic beads, and cell expansion was conducted in a wave bioreactor. Eleven days after activation, SAP UCAR-T cells were harvested, allocated, and processed for cryopreservation. After undergoing quality assurance (QA) and quality control (QC) testing, the cryopreserved cells were prepared for clinical trials.

We found that the CAR positive rate, cell viability, and proliferation were not affected by large-scale production (**Figures 8B–D**). Additionally, flow cytometry analysis confirmed that knockout efficiency was maintained during the scale-up process (**Figure 8E**). The proportion of memory phenotype SAP UCAR-T cells and the release of IFN-γ were also assessed, showing no significant changes between small-scale and large-scale production (**Figures 8F, G**). In summary, our pre-clinical studies demonstrate that SAP UCAR-T cells exhibit low immunogenicity, satisfactory persistence, and robust anti-solid tumor activity, along with reduced safety concerns both *in vitro* and *in vivo*. The stability observed during scale-up production further supports their potential for clinical application.

## Discussion

Despite its rapid advancement, the widespread application of CAR-T cell therapy is hindered by high costs and logistical challenges. Allogeneic CAR-T cells have emerged as a promising solution to these issues; however, their efficacy and safety still require enhancement (46). In this study, we successfully developed an allogeneic anti-CD70 CAR-T cell therapy using a triple gene knockout (tKO) strategy combined with the expression of a self-activated and protective (SAP) module. This approach effectively eliminates CD70-positive solid tumors while maintaining a favorable safety profile.

To minimize immune rejection faced by UCAR-T cells in the tumor microenvironment (TME), we overexpressed CD47, which enables tumor cells to evade immune surveillance (13), and has been shown the potential to protect CAR-T cells from clearance by macrophages and NK cells (24, 37). In our study, we found that the increase in resistance against macrophage rejection was more pronounced, suggesting that CD47 plays a more critical role in protecting UCAR-T cells from macrophages. In contrast, a more effective strategy is needed to mitigate damage from NK cells.

In addition to enhancing resistance to immune rejection, we also succeeded in improving the survival and efficacy of UCAR-T cells by activating IL-7 signaling (27, 47). IL-7 is a critical interleukin that promotes the survival, proliferation, and functionality of T cells primarily through the activation of IL-7Rα (48–50). By supplementing IL-7 during the *in vitro* expansion of CAR-T cells (51), constructing CAR-T cells that express IL-7 or IL-7R (36, 52), or treating mice with modified IL-7 following CAR-T infusion (53, 54), the persistence and anti-tumor efficacy of CAR-T cells can be significantly enhanced.

In this study, we combined the extracellular domain of CD47 with the transmembrane and intracellular domains of IL-7Rα to create the SAP module. Notably, a mutant form of the transmembrane domain of IL-7R was employed, allowing for the independent activation of IL-7 signaling in SAP UCAR-T cells, regardless of external IL-7 presence (36, 37). SAP UCAR-T cells exhibited superior survival and proliferation abilities both in an *in vitro* cytokine-free environment and *in vivo*. The SAP module also restored the anti-tumor activity of UCAR-T cells, which was alleviated probably due to the knockout of TCR and HLA molecules (55–57).

RNA-seq results revealed that compared with UCAR-T cells, chemokine receptor binding and chemokine signaling pathways were upregulated in SAP UCAR-T cells, suggesting enhanced infiltration into the tumor microenvironment and improved immune responses. This is consistent with the observed superior anti-tumor efficacy of SAP UCAR-T cells. In addition to well-characterized pathways, several less-studied genes may play pivotal roles in these enhanced therapeutic effects. For instances, in inflammatory bowel disease (IBD) research, proprotein convertase subtilisin/kexin type (PCSK6) has been implicated in increasing the proportions of Th1 cells and M1 macrophages (58), both of which are critical for effective immunotherapy. Interestingly, in our study, the knockout of TCR and HLA molecules led to a decrease in PCSK6 expression, while the SAP module restored its levels. This suggests that the SAP module may contribute to the therapeutic efficacy of SAP UCAR-T cells by upregulating PCSK6, thereby promoting Th1 cell differentiation and macrophage polarization. Another noteworthy gene is protein tyrosine phosphatase, non-receptor type 13 (PTPN13), which is known to induce apoptosis and restrict proliferation in tumor cells (59, 60). The decrease of PTPN13 in SAP UCAR-T cells may help to prevent apoptosis and improve cell survival, thereby enhancing their persistence *in vivo*. While these findings provide intriguing insights, further validation is required to elucidate the mechanisms of how the SAP module facilitates the UCAR-T therapy.

Despite the promising preclinical outcomes, we realize that several limitations need to be addressed to improve our SAP UCAR-T therapy. Firstly, the intensity of intracellular signaling pathways activated by the SAP module should be adjusted. Uncontrolled or excessively strong signaling could lead to T cell exhaustion or dysregulation, impacting both the effectiveness and safety of the therapy (61). Further optimization of the signaling cascades, such as introducing regulatory switches, may help better control signaling intensity and improve therapeutic outcomes. In addition to IL-7, other cytokines also showed beneficial effects on CAR-T cells (62), thus a more suitable signaling requires to be investigated. Another concern lies in the potential safety issues *in vivo*. The results in **Figure 4M** indicated that SAP UCAR-T cells could expand without the stimulation from tumor, therefore the tumorigenicity of SAP UCAR-T cells should be further confirmed.

In summary, we have successfully developed a CD70-targeting allogeneic CAR-T cell therapy, termed SAP UCAR-T, which demonstrated enhanced *in vivo* persistence and tumor-eliminating ability, along with minimal safety concerns in preclinical evaluations. Additionally, SAP UCAR-T cells displayed robust stability during scale-up production, an essential factor for upcoming clinical trials. SAP UCAR-T therapy holds promise as a strategic approach for treating a diverse patient population with CD70-positive solid tumors, offering lower costs and greater convenience.

## Materials and methods

### Cell lines

ACHN (renal cell carcinoma, CRL-1611), and CASKI (cervical cancer, CRM-CRL-1550) cells were purchased from ATCC and cultured in DMEM medium supplemented with 10% fetal bovine serum (FBS). U251 (glioblastoma, CL-0237) cells were obtained from Puno Sai and cultured in DMEM containing 10% FBS. BXPC3 (pancreatic cancer, CRL-1687) cells and 786-0 (renal cell carcinoma, CRL-1932) were sourced from ATCC and cultured in RPMI 1640 medium supplemented with 10% FBS.

### Flow cytometry analysis

The expression of CD70 CAR was analyzed using flow cytometry with FITC-Labeled Human CD27 Ligand/CD70 Protein and His Tag (ACRO, CDL-HF249). PE mouse anti-human CD70 antibody (BD#561935) was employed to detect the expression of CD70 on various cell lines. The phenotypes of CAR-T cells were analyzed using antibodies from BioLegend, including APC anti-human CD3 Antibody (317317), PE anti-human HLA-A,B,C Antibody (311405), FITC anti-human HLA-DR Antibod*y* (327005), APC anti-human CD47 Antibody (323123), PE anti-human CD4 Antibody (300507), APC anti-human CD8 Antibody (344721), APC anti-human CD69 Antibody (310909), APC anti-human CD45 Antibody (304011), APC anti-human CD45RO Antibody (304210), PE anti-human CD62L Antibody (304805), PE anti-human CD223 (LAG-3) Antibody (369205). The CytoFLEX flow cytometer (Beckman Coulter, Inc., California, USA) was then used to acquire flow cytometry data, and the data was analyzed using CytoExpert software.

### Construction of chimeric antigen receptors

The second-generation anti-CD70 CAR was designed to include a DNA fragment encoding an in-frame component from the 5’ end to the 3’ end: the CD8α signal peptide, anti-CD70 scFv, CD8 hinge region, CD28 transmembrane domain, and the intracellular co-stimulatory domains of CD28 and CD3zeta. This DNA fragment was codon-optimized using the GenSmart online tool, and synthesized by Anhui General Biotechnology Co., Ltd. (Anhui, China). The overall architecture of the plasmid originated from plasmid pCDH-CMV (Addgene, #72265), which was purchased from System Biosciences (USA). The SAP module was constructed by integrating the extracellular domain of CD47, the transmembrane domain of C7R (a mutant form of IL-7R), and the intracellular domain of IL-7Rα. The transmembrane domain of C7R used in this module is a mutant form (p.Thr244-Ile245insCysProThr) that can be activated independent of the presence of IL-7 according to previous studies (36). The SAP module was linked with the CAR structure by a T2A sequence.

### Jurkat reporter cell assay

The NFAT-Luc reporter Jurkat cell line was purchased from Jiman Biotechnology Co., Ltd. (Shanghai, China). Various CARs were transduced into the Jurkat cells using lentiviral vectors. Following transduction, the CAR-expressing Jurkat cells were co-cultured with 786-0 cells. The luminescence intensity of the Jurkat cells was measured using a plate reader, serving as an indicator of their activation level in response to CAR activation.

### Generation of CAR-T cells

CAR lentivirus was produced by transient transfection of HEK293T cells (CRL-11268, ATCC) using lentiviral plasmids. When the cells reached 80% confluence in T75 flasks, they were co-transfected with the lentiviral plasmid and packaging plasmids pMDLg/pRRE (Addgene, #12251), pRSV-Rev (Addgene, #12253), and pMD2.G (Addgene, #12259) using polyethylenimine (PolyScience, #24765-1). The medium was changed 48 hours post-transfection. Viral supernatants were harvested 48 and 72 hours after transfection, concentrated by ultracentrifugation at 22,000 rpm for 2 hours at 4°C, and stored at –80°C until use.

PBMCs were obtained from HeYou Biotech and MiaoShun Biotech and activated using anti-CD3/CD28 Dynabeads (Gibco) at a 1:1 bead-to-cell ratio in CTS™ OpTmizer™ T Cell Expansion SFM (Gibco), supplemented with 5% human platelet lysate, 5 ng/mL IL-7, and 10 ng/mL IL-15 (Miltenyi Biotec Technology & Trading Co., Ltd.). Twenty-four hours later, the activated T cells were transduced with the CAR lentivirus to generate CAR-T cells.

### Membrane proteome array screen

The membrane proteome array screen was conducted by Integral Molecular, Inc. (USA). In brief, plasmids containing cDNA clones of approximately 6,000 membrane proteins, representing over 94% of the human membrane proteome, were transfected into HEK-293T cells in 384-well plates, with each well containing 18,000 cells. This process generated membrane proteome array matrix plates. Test ligands were subsequently added to these matrix plates and incubated with the membrane protein-expressing HEK-293T cells. After incubation, the cells were washed with PBS and analyzed by flow cytometry using a fluorescence-labeled anti-CD70 antibody to identify the test ligand targets.

### CRISPR/Cas9-mediated knockout of TCR and HLA molecules

Two days post-CAR transduction, CRISPR/Cas9-mediated knockout of TCR and HLA molecules was performed. The gRNAs were mixed with Cas9 protein at a 5:4 ratio and incubated for 10 minutes. Following this, 1×10^6^ cells were electroporated using 20 μL buffer on a 4D-Nucleofector X-unit, according to the manufacturer’s instructions (Lonza). T cells treated with non-binding gRNA served as controls. After electroporation, the T cells were transferred to fresh medium and expanded at a density of 1×10^6^ cells/mL for 10 days.

### T7 endonuclease I assay

Genomic DNA was extracted from both unmodified and modified CAR-T cells, followed by PCR amplification targeting specific sites using the primers listed in **Supplementary Table S1**. The resulting amplicons were denatured and re-annealed to form heteroduplexes. T7 endonuclease I (T7E1; Nanjing Vazyme Biotech Co., Ltd.) was then used to digest these heteroduplexes, cleaving the mismatched DNA strands. The digested products were analyzed via gel electrophoresis to separate the resulting fragments.

### Detection of off-target rates of CRISPR/Cas9 system

Oligodeoxynucleotide (ODN) tags were introduced during the CRISPR/Cas9-mediated knockout of CAR-T cells, and DNA was extracted from the cells one day later. PCR amplification was conducted using forward and reverse ODN primers. The resulting PCR products were subjected to high-throughput sequencing, and the amplified sequences were compared with the corresponding target sequences.

### Measurement of CAR-T cytotoxicity

In the short-term killing assay, CAR-T cells and luciferase-expressing 786-0 (786-0-luc) cells were co-cultured at E:T ratios of 1:1, 3:1, and 9:1 for 6 hours. After incubation, the supernatants were removed, and tumor cells were treated with luciferase substrate for 10 minutes. The luminescence intensity was then detected and measured using a plate reader. The cell killing rate was calculated as [(background luminescence value - sample luminescence value)/background luminescence value] × 100%.

In the long-term killing assay, CAR-T cells were stimulated with 786-0-luc cells at an E:T ratio of 3:1. The CAR-T cells were then rechallenged with 786-0-luc cells for a total of four rounds. After each round, the phenotype and cytotoxicity of the CAR-T cells were assessed.

In the real-time cytotoxicity assay, a 96-well 20idf electrode plate was initially coated with 100 μL of 10 mM cysteine at 37°C for 4 hours. Following the removal of cysteine, the plate was washed twice with PBS, and 200 μL of tumor cells were seeded into each well. Once the target cells reached a plateau phase of growth, CAR-T cells were added at an E:T ratio of 1:1. Data collection continued until the end of the experiment.

### Detection of GVHD

PBMCs were isolated from three different healthy donors, irradiated with 20 Gy, and then combined. To distinguish between CAR-T cells and allogeneic PBMCs (allo-PBMCs) from the donors, the allo-PBMCs were labeled using the CellTrace CFSE Cell Proliferation Assay Kit (Thermo Fisher Scientific, C34570). CAR-T cells were co-cultured with allo-PBMCs at an E:T ratio of 1:1 for 24 hours. The expression of the activation marker CD69 on CAR-T cells was measured using flow cytometry analysis, while the apoptosis of allo-PBMCs was assessed using an apoptosis assay kit (Vazyme, A213-01).

### Detection of HVG reactions

PBMCs were isolated from three different healthy donors, and CD4^+^ and CD8^+^ T cells were separated using CD4 microbeads (Miltenyi, 130-097-048) and CD8 microbeads (Miltenyi, 130-045-201). The CAR-T cells were irradiated and labeled with CFSE. CD4^+^ and CD8^+^ T cells were co-cultured with various CAR-T cells separately at a 1:1 E:T ratio for 24 hours. The expression of CD69 on host T cells and the apoptosis of CAR-T cells were subsequently examined.

### Detection of cell apoptosis

Cells were washed with 1 ml of pre-chilled PBS and gently resuspended in 100 μL of binding buffer. Annexin V-PE and 7-AAD staining solutions were then added to the suspension, followed by incubation in the dark at room temperature for 10 minutes. Data were collected using flow cytometry.

### CD107a detection

NK cells were labeled with CellTrace CFSE and co-cultured with CAR-T cells at a 1:1 ratio in 200 μL of medium containing an anti-CD107a antibody. After 1 hour of co-culture, monensin was added to the medium at a concentration of 100 μg/mL, and the cell mixture was incubated for an additional 4 hours. After washing, the cells were resuspended in PBS and analyzed by flow cytometry. Degranulated NK cells were identified as the CFSE^+^ CD107a^+^ population.

### Macrophage phagocytosis detection

Macrophages and CAR-T cells were labeled with CellTrace CFSE and CellTrace Far Red, respectively. Macrophages were then co-cultured with target cells at a 1:1 ratio in 200 μL of medium for 6 hours. After washing, the cells were resuspended in PBS for flow cytometry analysis. Macrophages that phagocytized target cells were identified as the population double-positive for both fluorescent dyes.

### Western blotting

Protein samples were prepared by lysing cells with RIPA lysis buffer. The lysates were then boiled and denatured before separation by SDS-PAGE. Proteins were subsequently transferred onto a polyvinylidene difluoride (PVDF) membrane and incubated overnight with primary antibodies from Cell Signaling Technology, including phospho-STAT5 (Tyr694; #9359), phospho-BCL2 (Ser70; #2827), and β-Actin (#93473). After washing, the membrane was incubated with a secondary antibody for 1 hour, and protein expression levels were detected using autoradiography.

### Detection of cytokine release

Cell supernatants were collected during cytotoxicity detection assay and the levels of IL-6, IL-10, TNF-α, IFN-γ and Granzyme B secreted by CAR-T cells were assessed using the BD™ Cytometric Bead Array (CBA) Kit according to the manufacturer’s protocol.

### Animal experiments

Animal experiments were conducted in accordance with the regulations of the Animal Experiment Ethics Committee of Nanjing Normal University (IRB number: 2020-0047). To construct humanized immune system mice, 1×10^7^ PBMCs were injected intravenously via the tail vein into C-NKG mice (Cyagen, China) in a volume of 200 μL. Starting from the day of infusion, blood was collected from the tail vein every 7 days, and flow cytometry was used to determine the proportion of CD45^+^ cells, assessing the number of CAR-T cells.

For the xenograft models, 5×10^6^ U251 or 786-0 cells were subcutaneously injected into 6-week-old female C-NKG mice. When tumor volumes reached approximately 100 mm³, treatment was administered by intravenously injecting the indicated T cells in a volume of 200 μL. Tumor size, body weight, skin condition, fur quality, and posture of each mouse were continuously monitored. Mice were euthanized upon meeting predefined euthanasia criteria (significant weight loss, signs of distress) or as recommended by the veterinary staff. Tumors and other tissues, including heart, liver, spleen, lungs, kidney and brain were extracted.

Blood samples were collected 8 days post infusion. Genomic DNA was isolated from these samples using the DNeasy Blood & Tissue Kit (QIAGEN, 69506) following the manufacturer’s protocol. CD70 CAR copy numbers in genomic DNA were quantified via qPCR using the primers listed in **Supplementary Table S1**. To measure cytokine levels, mouse blood was left at room temperature for 20 minutes, centrifuged to obtain serum, and levels of IL-6, TNF-α, and IFN-γ were analyzed using the CBA assay.

### H&E and IHC staining

Mouse tissues were fixed with 4% paraformaldehyde (Boston BioProducts), embedded in paraffin, sectioned, and stained with H&E or indicated antibodies for IHC experiments. Images were obtained with a 3DHISTECH Panoramic digital slide scanner and the associated CaseViewer software (3DHISTECH).

### RNA sequencing and data analysis

PBMCs from the same donor were used to prepare CAR-T, SAP UCAR-T, and CD47 UCAR-T simultaneously. Cell pellets were collected and resuspended in TRIzol. RNA sequencing and subsequent analyses were performed by Genepioneer (Nanjing, China).

### Statistical analysis

All experiments were performed independently at least three times. Experimental data were analyzed and plotted using GraphPad Prism 8.0 software and are presented as means ± SD. The Student’s t-test was employed to compare differences between two groups, while one-way and two-way analysis of variance (ANOVA) were used for statistical analysis among multiple groups. Differences were considered statistically significant when p < 0.05: *p < 0.05; **p < 0.01; ***p < 0.001; ****p < 0.0001; ns, not significant.

## Data Availability

The original contributions presented in the study are included in the article/**Supplementary Material**. Further inquiries can be directed to the corresponding authors.
