# Supplementary material for: A self-activated and protective module enhances the preclinical performance of allogeneic anti-CD70 CAR-T cells
Source: Front Immunol. 2025 Jan 17;15:1531294. doi: 10.3389/fimmu.2024.1531294 (PMC11792090; doi:10.3389/fimmu.2024.1531294)
Supplement: Supplementary file 1 [file DataSheet1.docx]

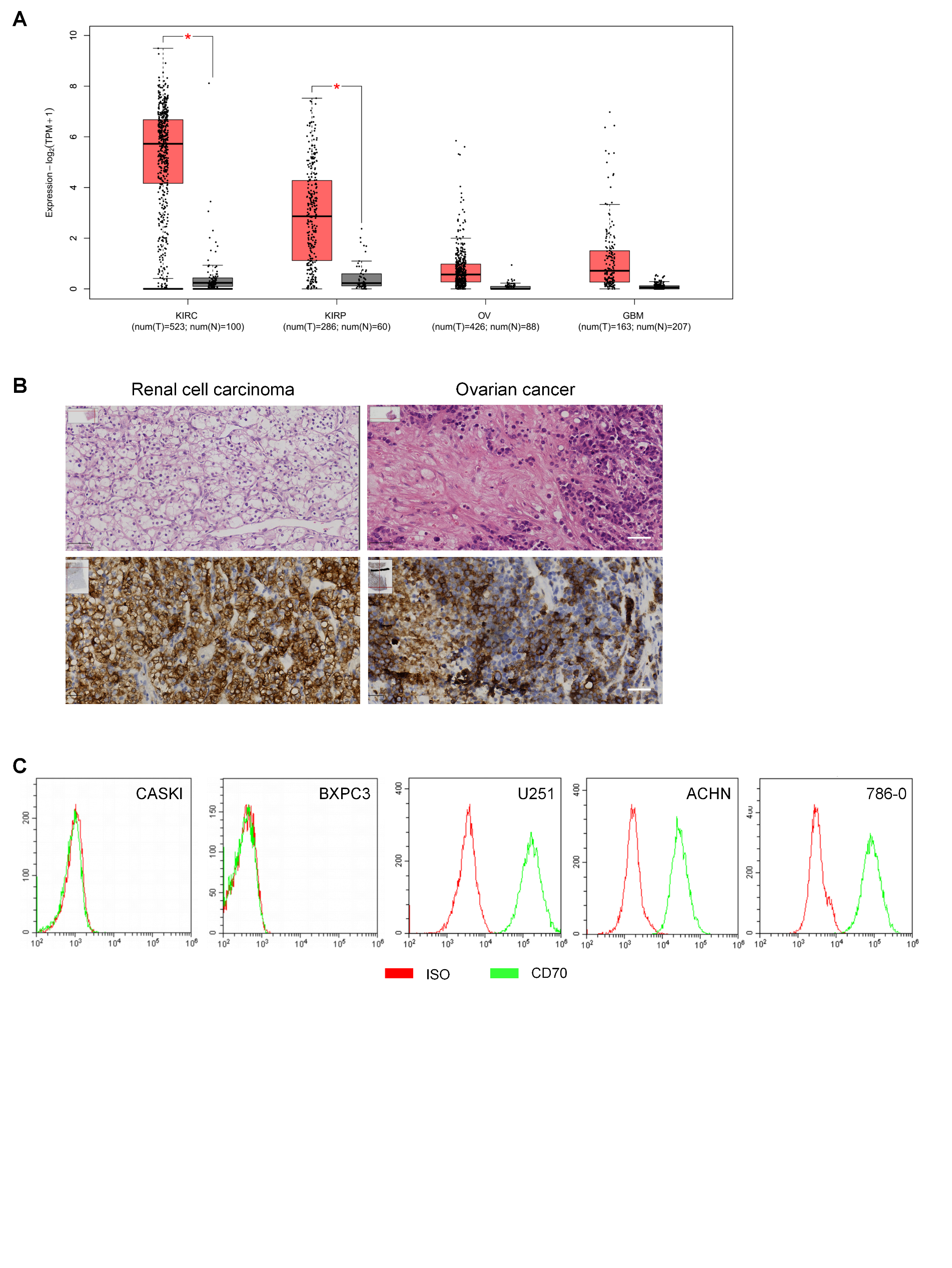


**Figure S1. High expression of CD70 in various tumors**

(A) Analysis of CD70 expression in tumor and normal tissues using the TCGA Database. (B) H&E and CD70 IHC staining of tumor tissues from patients with renal cell carcinoma and ovarian cancer. Scale bar, 50 μm. (C) Expression levels of CD70 in CASKI, BXCP3, U251, ACHN, and 786-0 cell lines.


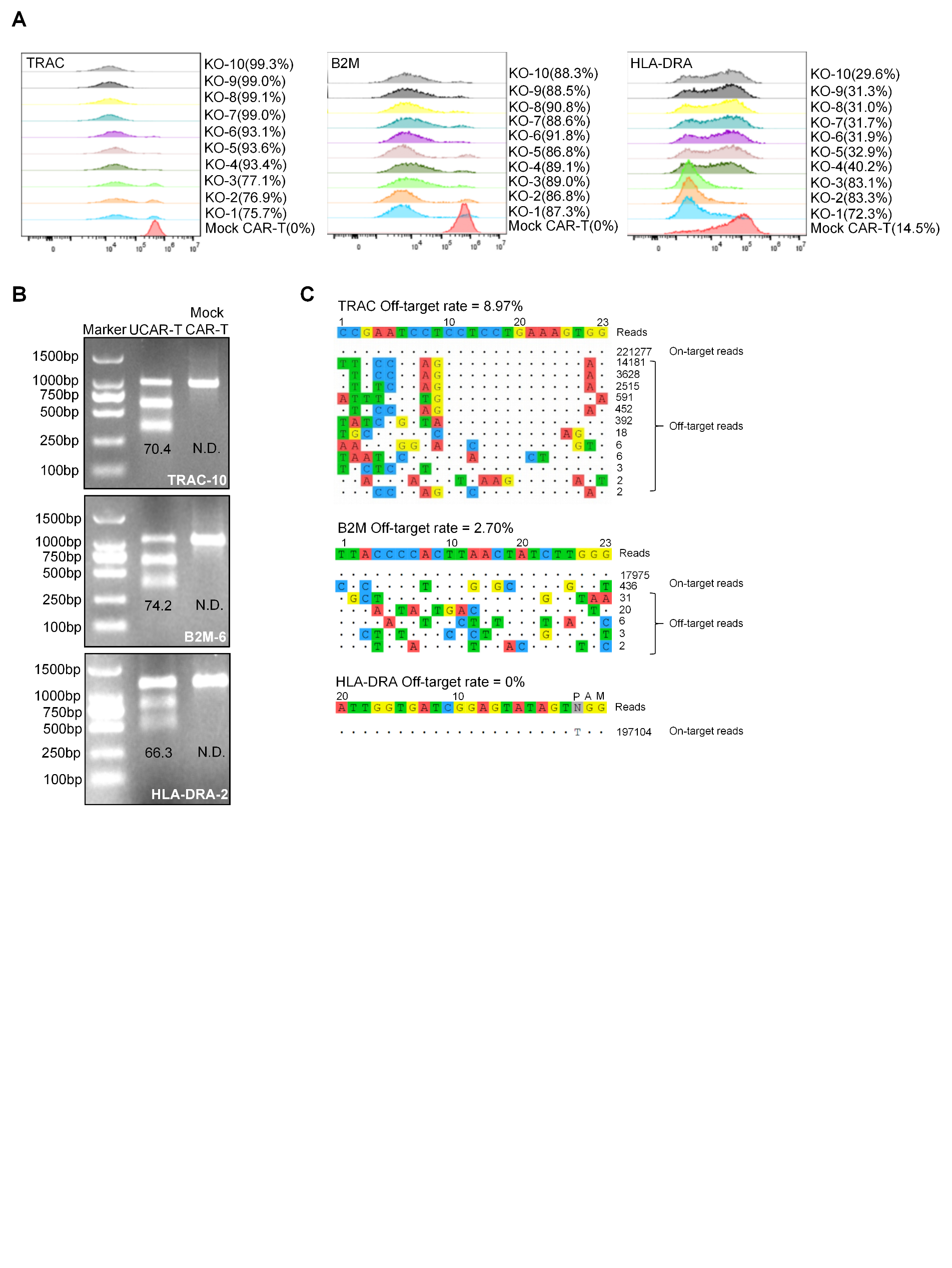


**Figure S2. Validation of TRAC, B2M and HLA-DRA knockout efficiency.**

(A) Flow cytometry analysis showing knockout efficiency using different sgRNAs. (B) T7E1 assay results verifying the gene editing efficiency of the sgRNA with the highest knockout efficiency from (A). (C) Guide-sequencing results indicating the off-target rates of sgRNAs.


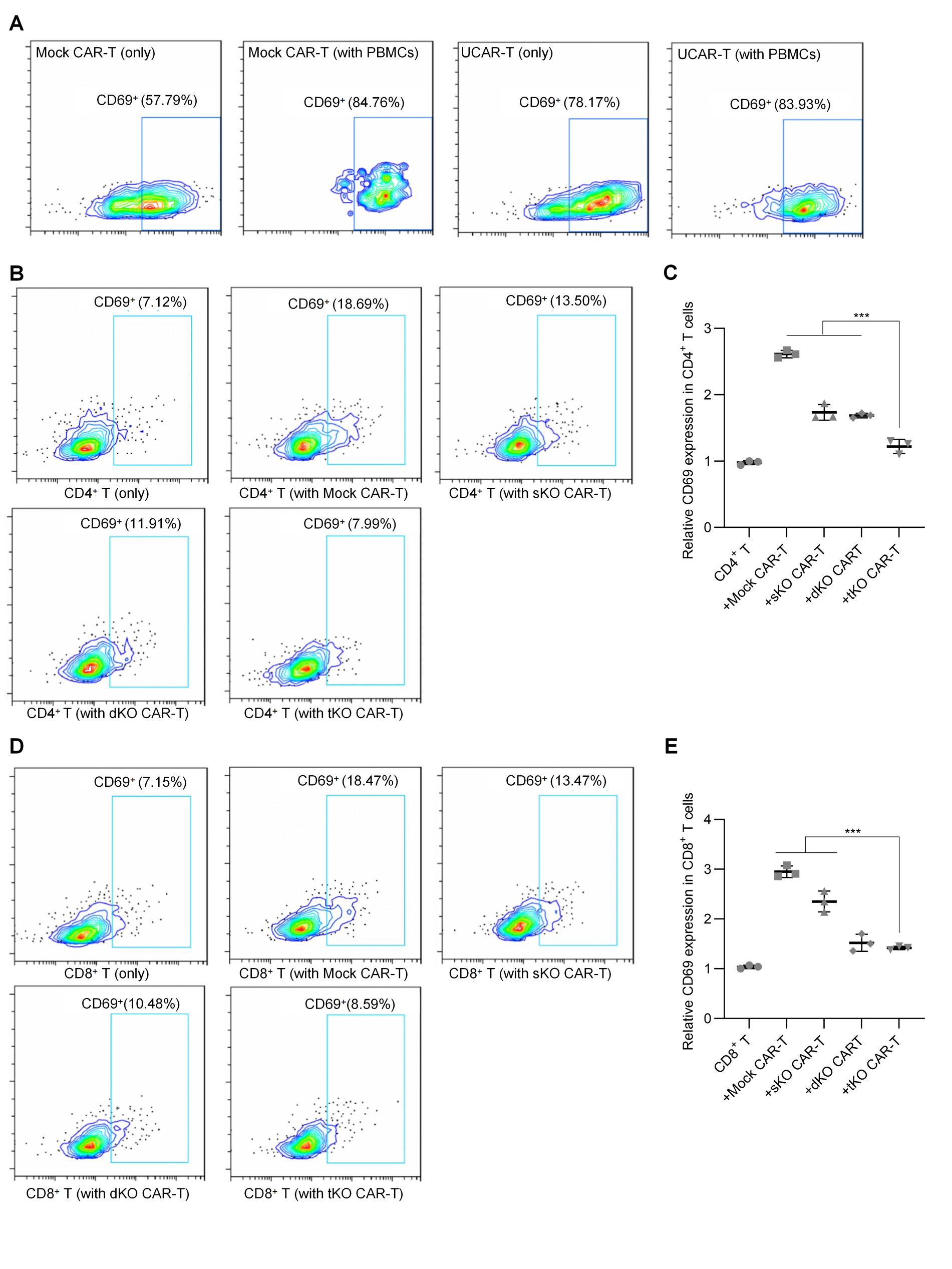


**Figure S3. Validating the GVHD and HVG reactions of UCAR-T cells**

(A) Representative flow cytometric profiles showing the expression of the activation marker CD69 on CAR-T cells with or without co-culture with PBMCs. (B and D) Representative flow cytometric profiles showing the expression of the activation marker CD69 on CD4^+^ (B) and CD8^+^ (D) T cells with or without co-culture with various CAR-T cells. sKO: CAR-T cells with TRAC KO; dKO: CAR-T cells with TRAC and B2M KO; tKO: CAR-T cells with TRAC, B2M, and HLA-DRA KO. (C and E) Quantification of relative CD69 expression in T cells co-cultured with various CAR-T cells. Data are means ± SD from at least 3 donors. Statistical significance was determined by one-way ANOVA. ***p < 0.001.


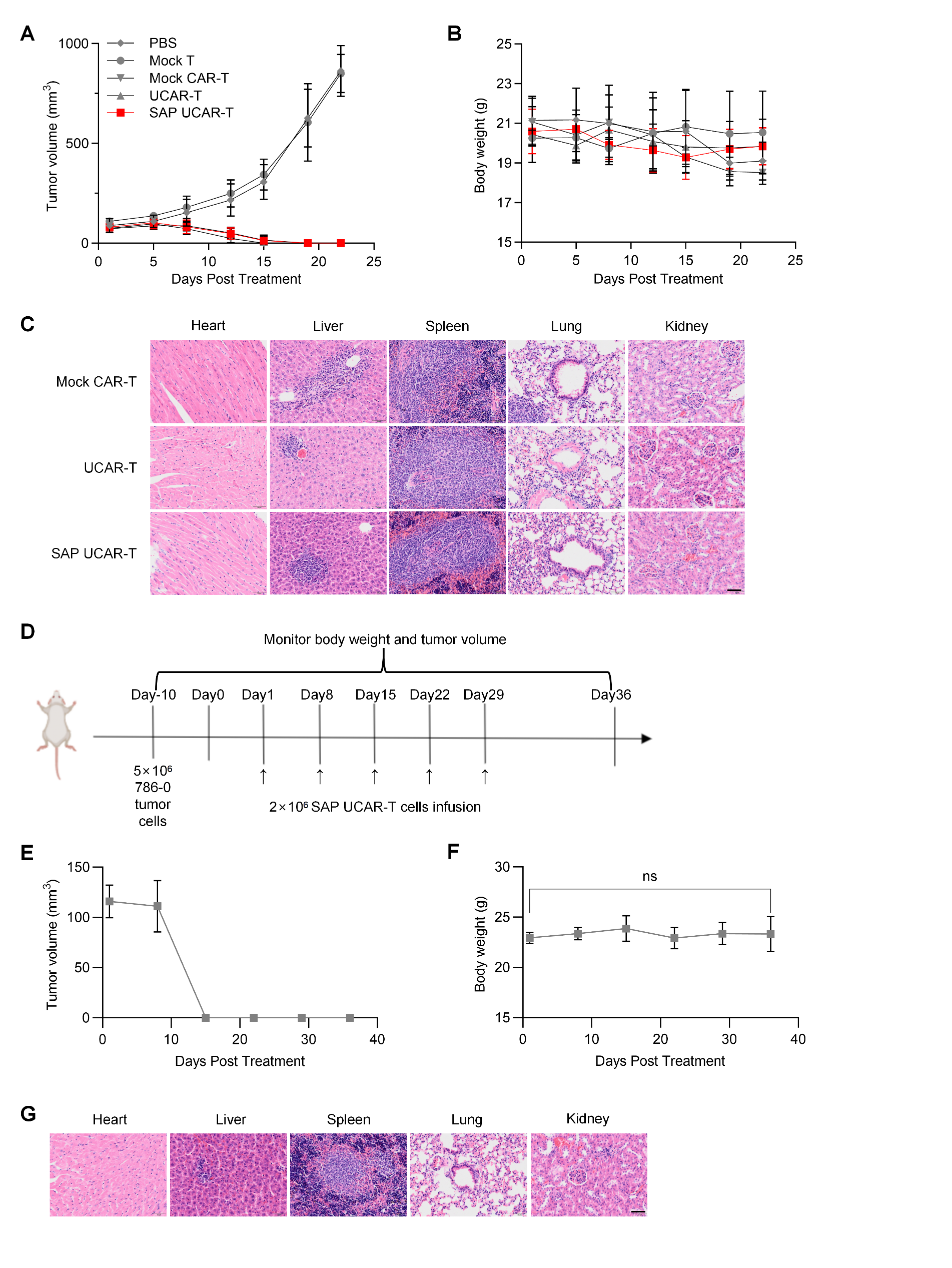


**Figure S4.** **High-dose and multiple CAR-T infusions did not raise safety concern in mice**

(A and B) Tumor volumes (A) and body weights (B) of mice receiving high-dose SAP UCAR-T infusions. (C) H&E staining to assess tissue damage caused by high-dose SAP UCAR-T infusion. Scale bar, 50 μm. (D) Mice were injected with SAP UCAR-T cells weekly for five times in total. (E and F) Tumor volumes (E) and body weights (F) of mice receiving multiple injections of SAP UCAR-T cells. (G) H&E staining to detect tissue damage resulting from multiple SAP UCAR-T cell injections. Scale bar, 50 μm. Data are presented as means ± SD. ns, not significant.

**Table S1.** **Primer sequences for Real Time PCR**

| Sequence Name | Primer sequence |
| --- | --- |
| TRAC-F | 5’GCCAAGTCTAGTCGGTGTTTCC3’ |
| TRAC-R | 3’ ACTCTAGCCAGGATTGGGGTG5’ |
| B2M-F | 5’CAGCCTGAAGTCCTAGAATGAG3’ |
| B2M-R | 3’AAAGTCTCCCCTGCTCCC5’ |
| HLA-DRA-F | 5’GGGAGCCAGAAGGGAAGT3’ |
| HLA-DRA-R | 3’CAGGAAGGGGAGATAGTGGA5’ |
| CD70 CAR-F | 5’AGAGACTTTGCTGCCTACAGAA3’ |
| CD70 CAR-R | 5’TTGTACAGCTGATTCTGCCCTT3’ |
